# Examining distractor qualities of pediatrics subject tests from a national assessment

**DOI:** 10.3389/fmed.2022.921719

**Published:** 2022-08-04

**Authors:** Qianqian Pan, Zhehan Jiang

**Affiliations:** ^1^Centre for Research in Pedagogy and Practice Office of Education Research, National Institute of Education, Nanyang Technological University, Singapore, Singapore; ^2^Institute of Medical Education, Peking University, Beijing, China; ^3^National Center for Health Professions Education Development, Peking University, Beijing, China

**Keywords:** reliability, psychometrics, pediatrics, distractor, assessment

## Abstract

**Background:**

Analyzing distractor qualities of a pediatrics subject test in a national-level examination is vital in developing high-quality items for the discipline. Yet traditional approaches focus on key answers only and therefore are less informative. The number of distractors can also be parsimonized to improve the item development.

**Materials and methods:**

From a pediatrics subject test at the national level, raw responses of 44,332 examines to nineteen multiple-choice questions were analyzed, such that the distractor qualities were evaluated *via* traditional and advanced methods such as canonical correlation index. Additionally, a simulation study was conducted to investigate the impact of eliminating distractor numbers on reliability.

**Results:**

The traditional item analysis showed that most items had acceptable psychometric properties, and two items were flagged for low item difficulty and discrimination. Distractor analysis showed that about one-third of items had poorly functioning distractors based on relatively a low choice frequency (<5%) and a small effect size of distractor discrimination. The simulation study also confirmed that shrinking distractor numbers to 4 was viable.

**Conclusions:**

Integrating multiple methods, especially the advanced ones, provides comprehensive evaluations of the item quality. Simulations can help re-consider the decision to set distractor numbers for cost-effectiveness. These proposed methods can improve further development of the pediatrics subject test.

## Introduction

A well-designed assessment is beneficial for accurately evaluating whether educational goals or standards are being met and perhaps can shape the education in the aspects of teaching practice, placement criteria, and other related policies (1); the stakes are heavily enlarged when it comes to examination at the national level. Many nationwide standardized assessments for general physicians' list pediatrics have a must-have discipline in the test domains. For example, the United States Medical Licensing Examination and the National Medical Licensing Examination in China deliver items attributed to the pediatrics discipline. The subject test results from national assessments of this kind are informative to the mastery of general pediatrics knowledge and the potential prediction of future board passage in the area (2).

However, compared to other disciplines, pediatrics discipline's item proportions are generally lower. Ensuring high qualities for the restricted shares becomes critical in reliably and validly measuring the pediatrics knowledge. Traditionally, when it comes to item quality insurance, most analyses focus on keys (i.e., correct answers) only, resulting in an overemphasis on the difficulty index and the discrimination index. Specifically, the difficulty index counts the proportions of examinees answering the question correctly, whereas the discrimination index compares the assessment results of high sum-score groups with that of low total-score groups (3). This practice, however, neglects the properties of distractors, which are meant for effectively drawing examinees away from the key (4). For any rigorous test providers, analyzing the qualities of distractors is essential to multiple-choice exams [(5, 6); Multiple-choice questions (MCQs)].

Ideally, a distractor displaying adequate discriminatory power related to the target construct (e.g., medical knowledge) should be selected by enough examinees. Generating high-quality distractors is undoubtfully expensive, and the cost is amplified when it comes to the assessments in medical education (7). Further, item developers often find it extremely challenging to write good distractors (8). Therefore, understanding the characteristics of distractors is necessary for a comprehensive test-quality evaluation and informative decisions about future improvement.

In this article, we collected and described items from a pediatrics subject test assembled in the Standardized Competence Test for Clinical Medicine Undergraduates (SCTCMU), which provides summative results for undergraduate education and differentiates itself from its nation's licensing exam. The item distractors were then evaluated *via* both traditional and novel approaches. We conducted a simulation study to refine the number of distractors in the assessment based on the distractor analysis.

## Methods

### Participants and items

The pediatrics subject test consisted of 19 items extracted from the SCTCMU, a national assessment administered to students before their clerkship in China. The 19-item dataset includes the specifications (e.g., Bloom's taxonomy levels), the MCQs' answer keys, and the original responses of all 44,332 examinees (i.e., the choice distributions in all MCQs' five options). These items covered nine knowledge nodes, including Treatment principles, Basic clinical concepts, Disease prevention and rehabilitation, Etiology and pathogenesis, Diagnosis and differential diagnosis, Auxiliary examinations, Basic medical knowledge, Clinical manifestations, and Basic medical knowledge. All items were in multiple-choice format with five answer options and a single key.

### Item analyses

Item analysis is crucial to providing a source of validity to support the validity argument (9). Essentially, investigating items' properties lies within the concept of validity. The proposed approach in this study unsurprisingly utilized for completing the item analysis step within the validation process, however, broadens the methodological view. Traditional analysis usually includes items' difficulties, item discrimination, and test reliability (e.g., Cronbach's alpha). Explicitly, item difficulty is defined as the percentage correct (or *p*-values), the percentage of examinees who got the item correct in the sample. Item discrimination indices include the item-total correlation (RIT; point-biserial correlations between the item and total scores) and the item discrimination index (ULI; the difference in the ratios of correct answers in the upper and lower thirds of examinees). More importantly, we evaluated the distractor quality by (1) calculating the distractor choice frequency, the point-biserial correlation, and rising selection ratio, as well as plotting the option trace line plot, and (2) computing the effect sizes to detect discriminatory distractors using the average canonical correlation of each item (*R*_*CC*_).

#### Distractor analysis

##### Distractor choice frequency

One commonly used distractor evaluation rule, the distractor choice frequency, was examined first. Traditionally, the distractor with a choice frequency lower than 5% (*P*_*dj*_ < 0.05) would be regarded as non-functioning and need revision (8, 10). However, in recent years, researchers argued that this method did not account for the dependency between item difficulty and distractor as one limitation since the threshold of 0.05 was not sensitive to the easy item (11). Especially, in the professional credentialing tests (i.e., examinations for physician licensure), most examinees are expected to master most items, such that the rates of non-functional distractors might be exaggerated (12). To overcome this limitation, Raymond et al. (4) proposed using a non-constant threshold to evaluate the non-functional distractors, considering the item difficulty. As shown in Equation (1), *P*_*c*_ is the percentage correct of one item and *P*_*dj*_ is the frequency of one distractor, then the threshold of a non-function distractor is set as *P*_*df*_ < *P*_*nfd*_. Obviously, this threshold is varied across items, where easier items will have smaller *P*_*nfd*_ than harder items. Results from analyzing a large item pool for a physician licensure examination supported using this new threshold over the traditional one with 0.05:


(1) $$\begin{array}{r} P_{ndf}=0.1-\left(P_{c}\;*\;0.1\right) \end{array}$$

##### The point-biserial correlation

Well-functioning distractors are supposed to show a negative point-biserial correlation (*PB*_*D*_) (13). However, a previous study showed *PB*_*D*_ did not provide useful information for developers in some situations, for example, difficult items might have positive *PB*_*D*_ values, even in the distractors function. So, we adopted an alternative index *PB*_*DC*_ proposed by Attali and Fraenkel (13) to overcome the limitations of *PB*_*D*_, which contrasts the group who chose one distractor only with the group who solved the item. Then, the average of *PB*_*DC*_ across all possible distractors of each item was computed to examine whether selecting the key is a function of ability levels while comparing to selecting distractors. Typically, the lower the average *PB*_*DC*_ value is, the better the quality of distractors is. In general, below −0.30 is recommended.

##### Rising selection ratio

Rising selection ratios represent the odds of choosing the correct option vs. a distractor, which should be a monotone increasing ability function. This study adopted Goodman and Kruskal's γ for evaluating the rising selection ratio suggested by previous studies (14, 15). The higher value of γ is, the better the average quality of distractors is, and a simulation study suggested using 0.3 as the threshold (10).

##### Option trace line plot

**Figure 1:**
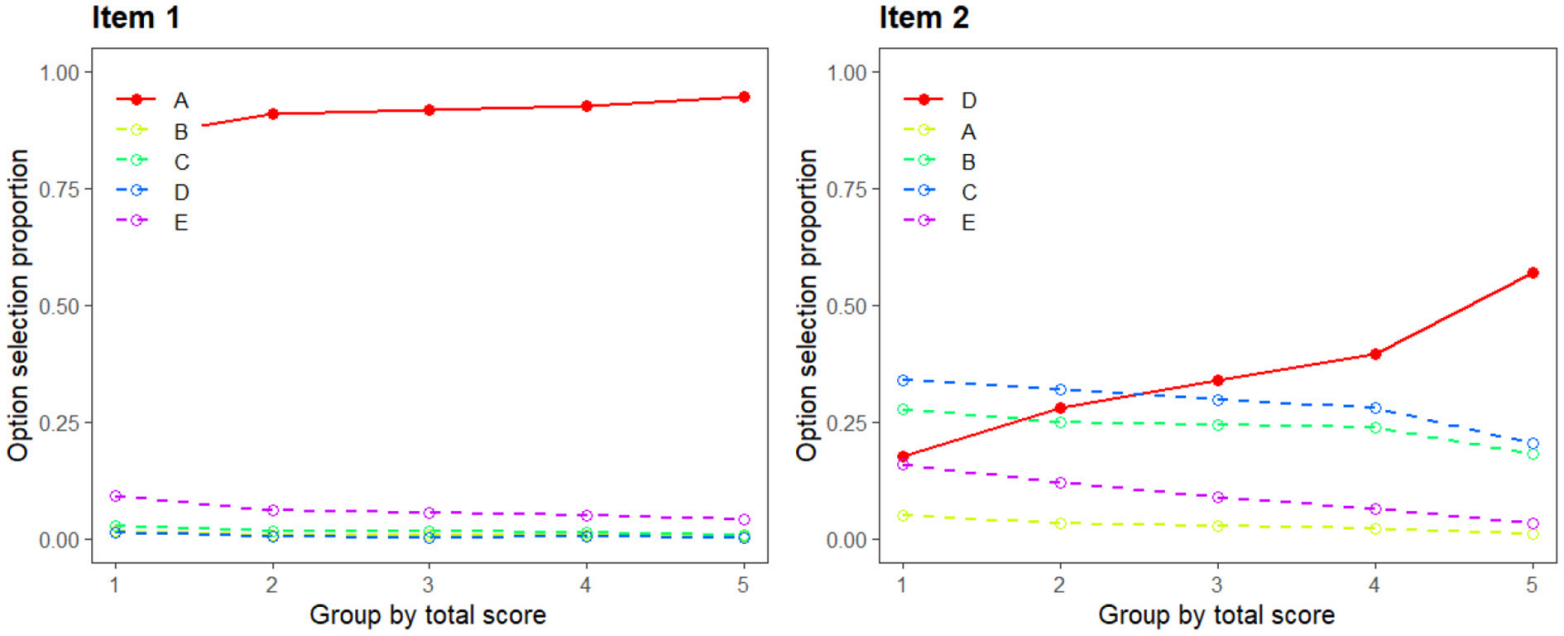
Trace line plots of two items.

Option trace line plot is another normally used in graphical way to present the relationship between the frequency of choice and the ability levels of examinees (11, 16). Figure 1 displays two trace plots, where the x-axis represents five levels of examinees' ability based on the total score, the y-axis represents the option selection proportion, the solid line presents the answer key, and other lines represent the distractors. The optimal trace lines would show that the frequency of choosing the correct option is a positive function of the total score; meanwhile, the frequency of choosing the distractors decreases as the total score increases. Therefore, Item 2 has four well-functioning distractors compared to Item 1.

#### Effect sizes for the detection of discriminatory distractors

We computed the average canonical correlation of each item (*R*_*CC*_) to evaluate the distractor effect size. As one type of multivariate analysis method, the canonical correlation could be used to describe the relationship between one multivariate set of variables (i.e., a matrix of distractor choices of each item) and one continuous variable (i.e., the total scores of examinees who did not solve the targeted item), indicating the discriminatory power of distractors (17). A previous simulation study suggested that the canonical correlation combined with a 0.30 threshold for pre-selecting items (10). Considering the page limits and the focus of the current study, more details of *R*_*CC*_ can be found in Forthmann and colleagues' study (10).

#### A simulation study on eliminating distractors

We further studied the effect of eliminating one distractor on item parameters and test scores. We adopted a two-step procedure. Firstly, three methods were applied to eliminate one distractor and then assign one answer option to examinees who selected these eliminated options, including (1) eliminating the least popular distractor and randomly assigning one of the rest answer options; (2) randomly eliminating one distractor and randomly assigning one of the rest answer options; (3) randomly eliminate one distractor and then assign answer options according to the original proportion of rest answer options. Similar methods have been employed in previous students [e.g., (4, 18, 19)].

After the elimination process, reliability α was computed based on the new data. In addition, a three-parameter logistic (3PL) item response theory (IRT) model was fit to the new data to estimate item parameters and individual scores using the expected a posteriori (EAP) method and both empirical and marginal reliability of the estimated score. The same elimination process and estimation process were repeated 100 times and were further applied to the SCTCMU pediatrics subject test.

## Ethics

### Ethics committee approval

Ethical review and approval were not required for the study on human participants in accordance with the local legislation and institutional requirements.

### Consent procedures

Written informed consent from the junior doctors working at Flinders Medical Center was not required to participate in this study in accordance with the national legislation and the institutional requirements.

## Results

The results of the traditional item analysis are presented in Table 1: the difficulty (i.e., proportion correct) normally spreads with the central point at 0.54 and the standard deviation at 0.22. Conventionally, items with a discrimination estimate smaller than 0.2 should be flagged as possible low quality. Meanwhile, lower discrimination co-occurs in extremely easy or difficult items (20). The phenomenon reflects in Items #1 and #15 as they were relatively easy, and discriminations fell below the conventional threshold. Overall, the 19-item set produces 0.605 of Cronbach's α, which was a little lower than the common rule of thumb of 0.7 [e.g., (21, 22), p. 28]; however, only a total of 19 items were included in the current analysis; therefore, the limited number of items might lead to a low Cronbach's α (23).

**Table 1:** Traditional item analysis results.

**Item ID**	***P*_*c*_**	**SD**	**RIT**	**ULI**	**α.drop**
1	0.91	0.29	0.12	0.07	0.61
2	0.34	0.47	0.30	0.30	0.60
3	0.39	0.49	0.33	0.36	0.60
4	0.86	0.35	0.28	0.21	0.60
5	0.88	0.32	0.29	0.20	0.60
6	0.83	0.38	0.28	0.23	0.60
7	0.36	0.48	0.24	0.25	0.61
8	0.42	0.49	0.38	0.42	0.59
9	0.54	0.50	0.43	0.49	0.58
10	0.58	0.49	0.29	0.32	0.60
11	0.72	0.45	0.39	0.39	0.59
12	0.40	0.49	0.54	0.62	0.56
13	0.17	0.37	0.41	0.31	0.58
14	0.49	0.50	0.45	0.50	0.58
15	0.78	0.41	0.19	0.16	0.61
16	0.44	0.50	0.54	0.62	0.56
17	0.35	0.48	0.51	0.55	0.57
18	0.61	0.49	0.22	0.21	0.61
19	0.64	0.48	0.36	0.38	0.59

*P_c_, item difficulty estimated as an average item score divided by its range; SD, standard deviation; RIT, Pearson correlation between item and total score; ULI, Upper-Lower Index; α drop, Cronbach's α of test without given item (Cronbach's α for the test as a whole is provided in the note below the table)*.

### Distractor analysis results

Firstly, we examined the distractor choice frequency(*P*_*df*_) and flagged the distractors by applying two criteria (1) *P*_*df*_ <5% and (2) *P*_*df*_ < *P*_*nfd*_ (See Equation 1). As shown in Table 2, the criteria of *P*_*df*_ < *P*_*nfd*_ flagged fewer items than the criteria of *P*_*df*_ <5%, which is coherent with the nature of the licensure test. Meanwhile, a total of 17 out of 19 items had at least one non-functioning distractor regardless of criteria, indicating the possibility of removing non-functional distractors. More specifically, after considering the item difficulty, Item 10 had three distractors with relatively low choice frequencies. There are seven items that had been flagged for two unfunctional distractors.

**Table 2:** Distractor analyses results.

**Item ID**	**Number of Distractors with Relative Choice Frequency < 0.05**	**Number of Distractors with Relative Choice Frequency < p_nfd_**	**PB_DC_**	**γ**	**R_CC_**
1	3	2	−0.11	0.22	–
2	1	1	−0.26	0.62	0.25
3	2	2	−0.21	0.46	0.13
4	3	2	−0.20	0.62	–
5	3	1	−0.21	0.70	–
6	2	1	−0.18	0.63	0.23
7	1	2	−0.19	0.38	0.24
8	1	1	−0.35	0.68	0.20
9	1	1	−0.31	0.59	0.44
10	3	3	−0.18	0.38	–
11	2	0	−0.27	0.63	0.28
12	2	2	−0.47	0.75	0.30
13	1	1	−0.43	0.65	0.22
14	2	2	−0.29	0.65	0.32
15	1	0	−0.12	0.39	0.06
16	1	2	−0.45	0.76	0.31
17	1	1	−0.48	0.65	0.28
18	1	1	−0.14	0.37	0.33
19	1	0	−0.21	0.62	0.25

*R_CC_ is not calculated when the number of distractors with relative choice frequency <0.05 exceeded a value of five (i.e., when only one distractor remained for analysis)*.

In addition, the average *PB*_*DC*_ of each item was computed, which represents the average contrast between the group selecting the key and the group selecting distractors. As suggested by a previous study, the cutoff value was set to −0.3, meaning items with the average *PB*_*DC*_ ≤ −0.3 were regarded as having well-functioning distractors. As shown in Table 2, there are about one-third of items having well-performed distractors (Items 8, 9, 12, 13, 16, and 17) in the current data.

**Figure 2:**
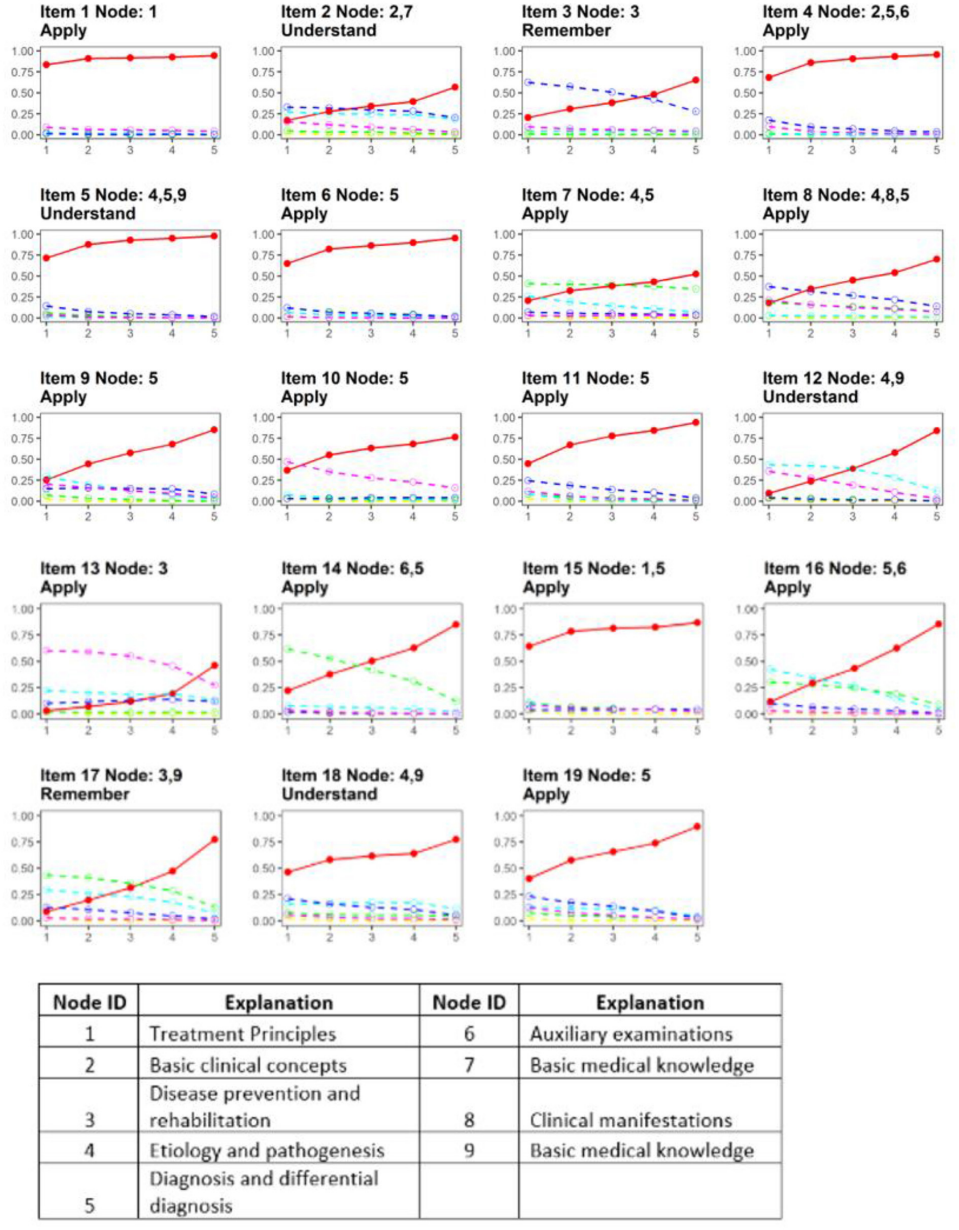
Trace line plots of all items with assessed nodes and cognitive levels.

The average γ values for all available distractors of each item were computed, and the results showed Item 1 was flagged for insufficient the rising selection ratio property. In addition, trace plots of all items are presented in Figure 2 to show the rising selection ratio directly, where the y-axis represents the percentage of participants and the x-axis presents five groups based on the total score^1^. The solid red line represents the key, while the other lines represent the distractors. Such that the red line is expected to monotonically rise with the total scores increasing. Meanwhile, the other lines are expected to be separated from each other and decrease. In addition, the node and Bloom's taxonomy level of each item are also shown: the majority of items demonstrates sufficient discrimination power. Moreover, we can find that Items 3, 8, 12, 13, 14, 16, and 17 had well-functioning distractors. However, the frequency of choosing the key and distractors was almost consistent across different groups in Items 1, 4, 5, 6, and 15, indicating the low discriminatory power of options and room for improvement. Among items with poorly performed distractors, three items were at the Apply level of Bloom's taxonomy. In addition, all of them were assessing Nodes 1 and/or 5. These findings might reveal particular challenges in developing high-quality distractors for items assessing the “Treatment principles” and “Diagnosis and differential diagnosis” at the application level.

In addition, the distractor effect size was measured by the average canonical correlation of each item (*R*_*CC*_). A previous simulation study suggested that the canonical correlation combined with a 0.30 threshold for pre-selecting items (10). Results found that Items 9, 12, 14, 16, and 18 had medium effect sizes to detect discriminatory distractors (canonical correlation ≥ 0.3). In the meantime, more than two-thirds of items had relatively low discriminatory power.

In sum, these three indices focused on different aspects of discrimination of distractors, where *PB*_*DC*_ and γ focused more on the relationship between examinees' ability and solution behaviors and less on the contrast among distractors. Meanwhile, *R*_*CC*_ focuses more on the contrast among distractors and provides the effect size of distractors. Therefore, these results did not covary, but they could complement each other and provide us a relatively comprehensive review on developing answer options. Together, these results reveal that about one-third to half of the items has relatively well-functioning distractors, and the distractors of the rest of the items might need some revisions.

### Effects of eliminating one distractor

Table 3 displays the results from the simulation study on investing the effects of eliminating one distractor from the original data. As expected, eliminating one option reduced both marginal and empirical reliability and item difficulty and increased the guessing parameter. The most notable changes happened in item difficulty parameters; items became easier on average, especially under Method 3. However, the changes in reliability were negligible. Furthermore, the aggregated correlation between estimated scores between the original, and the new data were close to 1. These results are consistent with previous studies (4). Regardless of distractor elimination methods, the test reliability will not be negatively impacted.

**Table 3:** Test reliability, test scores, and item parameter estimates after eliminating distractors.

**Method of elimination**	**Method of Assignment**	**α**	**Marginal reliability**	**Empirical reliability**	**a**	**b**	**c**
No elimination		0.560	0.693	0.696	1.113	−0.478	0.111
Method 1: Eliminated the least popular distractor	Random assignment	0.568	0.662	0.669	1.198	−1.201	0.190
Method 2: Randomly eliminated one distractor	Random assignment	0.591	0.650	0.656	1.143	−1.379	0.199
Method 3: Randomly eliminated one distractor	Original proportion	0.629	0.623	0.628	1.094	−3.055	0.231
	**Factor scores after elimination**						
	Method 1	Method 2			Method 3		
Original Factor scores	0.991	0.977			0.950		

*a, b, and c represent the item discrimination, item difficulty, and guessing parameters from a unidimensional 3PL IRT model*.

## Discussion

This investigation is based on national assessment data in China, where the raw responses in the Pediatrics discipline were collected and analyzed. Overall, it can be seen that the majority of the selected items were developed appropriately, as the distractors demonstrated good discriminatory power according to the rules of thumb.

We conducted the distractor analysis, which revealed that some distractors did not perform well-based on the relatively low choice frequency with/without considering the item difficulty along with a small effect size of distractor discrimination. In addition to examining the psychometric properties, the results revealed some challenges in developing high-quality distractors for items assessing the “Treatment principles” and “Diagnosis and differential diagnosis” at the application level.

In addition, the distractor analysis results indicated the possibility of eliminating some non-functioning distractors. Then, we conducted a small simulation to explore the effects of eliminating one distractor under three conditions because most items had at least one non-functioning distractor. Previous studies suggested that a four-option multiple-choice item was less influenced by test-taking strategies and easier to balance the option set (24). The results confirmed that eliminating one distractor did not negatively impact the test reliability nor increase the guessing parameters, which are aligned with previous studies [e.g., (4, 25)]. Together, it suggests the possibility of developing items with four answer options in future assessments, which reduce the cognitive burden of test developers to some degree without lowering the test reliability (i.e., more cost-effective).

It should be noted that this article does have limitations. Firstly, the evaluation was purely psychometric-based; in a high-stake setting, changes should almost always be verified by subject matter experts and stakeholders. Secondly, the internal connections among different disciplines were absent for the article, making the inferences less generalizable to the entire test. Last but not least, although the thresholds used in the analysis were proposed after symmetric studies, misclassifications such as type-I errors are still inevitable.

## Conclusions

Integrating multiple methods, especially the advanced ones, provides a comprehensive evaluation of item quality. Simulations can help reconsidering the decision on setting distractor numbers for the sake of cost-effectiveness. These proposed methods can improve the further development of the pediatrics subject test.

## Data availability statement

The original contributions presented in the study are included in the article/supplementary materials, further inquiries can be directed to the corresponding author/s.

## Ethics statement

Ethical review and approval was not required for the study on human participants in accordance with the local legislation and institutional requirements. Written informed consent from the (patients/participants OR patients/participants legal guardian/next of kin) was not required to participate in this study in accordance with the national legislation and the institutional requirements.

## Author contributions

ZJ and QP proposed the idea together, while QP handled both the design and the data analysis, and ZJ conducted the literature review and the discussion. All authors contributed to the article and approved the submitted version.

## Funding

This work was supported by National Natural Science Foundation of China for Young Scholars under Grant 72104006; Peking University Health Science Center under Grant BMU2021YJ010.

## Conflict of interest

The authors declare that the research was conducted in the absence of any commercial or financial relationships that could be construed as a potential conflict of interest.

## Publisher's note

All claims expressed in this article are solely those of the authors and do not necessarily represent those of their affiliated organizations, or those of the publisher, the editors and the reviewers. Any product that may be evaluated in this article, or claim that may be made by its manufacturer, is not guaranteed or endorsed by the publisher.

## Footnotes

^1^Group score distribution: group 1: [0,4], group 2: [4,8], group 3: [8,12], group 4: [13,16], group 5: [17,19].
